# Review and Publication Times and Reporting Across Journals on Health Policy

**DOI:** 10.1001/jamanetworkopen.2025.12545

**Published:** 2025-05-27

**Authors:** Kathryn A. Phillips, Danea M. Horn

**Affiliations:** 1 Center for Translational and Policy Research on Precision Medicine, Department of Clinical Pharmacy, University of California, San Francisco; 2 Institute for Health Policy Studies, University of California, San Francisco

## Abstract

This cross-sectional study compares how a broad range of journals vs a narrow set of specialty journals report and measure review and publication times and assesses these patterns across journals.

## Introduction

The move toward open access publication has focused attention on peer-reviewed academic journals’ publication timelines.^1^ This focus is particularly true for journals publishing health policy research, for which rapid dissemination could facilitate evidence-based policy decisions.^2,3^ We examined how these journals report and measure their review and publication times (RPTs) and assess patterns across journals. Our aim was to include a broad range of journals vs a narrow set of specialty journals to enhance generalizability and focus on journals vs articles as the unit of analysis,^4,5,6^ enabling visibility into RPT reporting.

## Methods

This cross-sectional study used publicly available data; thus, institutional review board review and informed consent were not required in accordance with the Common Rule. The study followed the STROBE reporting guideline.

 Our study included journals that publish health policy research (including influential general interest journals given their impact on dissemination) and have an h-5 index greater than 23 and impact factor of 2 or higher. Final inclusion was based on review by 3 journal editors (eTable in Supplement 1). We examined 6 metrics as of November 18, 2024, and used descriptive analysis to assess RPT reporting and evaluate differences between journal characteristics using the 2-sided (*P* < .05) Wilcoxon rank sum test. Data were analyzed using Stata, version 18 (StataCorp LLC).

## Results

Our sample of 57 journals included 25 open access and 32 hybrid/subscription journals. Six journals were highly selective (high impact factors and low acceptance rates), 9 (16%) did not report RPTs, but 21 (37%) reported 3 or more metrics (Figure). The complete publication process varied from 35 to 353 days (Table). Time to first decision (without peer review) and article acceptance to publication time were most reported. Median (range) time to first decision before peer review was 10.0 (2.0-67.0) days; to first peer-reviewed decision, 60.5 (21.0-263.0) days; and to final peer-reviewed decision, 198.0 (38.0-314.0) days. Median (range) time from acceptance to online publication was 25.5 (2.0-205.0) days.

**Figure.**
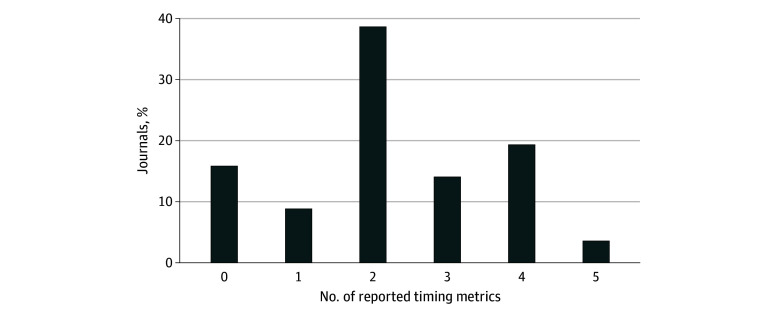
Percentage of Peer-Reviewed Academic Journals Reporting Review and Publication Timing Metrics Review and publication timing metrics were collected from each journal’s (N = 57) primary website or partner website if the journal was owned by a larger publisher. If review and publication timing metrics were reported in 2 places (ie, a year-in-review article, on the About or For Authors page), the metrics listed on the standard webpage were prioritized. If the journal reported a range of timing for a metric, the median time was used in the analysis. All review and publication timing metrics were double-entered, and data discrepancies were resolved by consensus between investigators (K.A.P. and D.M.H.).

**Table.** RPTs for Academic Journals That Reported at Least 1 Review and Publication Timing Metric (n = 48)^a^

Publication timing	No. of journals	RPT, d		*P* value
		Mean (SD)	Median (range)	
**Time-to-first decision comparisons**				
All journals	38	13.9 (12.8)	10.0 (2.0-67.0)	NA
Open access	16	16.1 (16.5)	13.0 (3.0-67.0)	.60
Hybrid/subscription	22	12.3 (9.3)	9.5 (2.0-31.0)	
Highly selective	5	4.6 (2.7)	3.0 (2.0-8.0)	<.001
All others	33	15.3 (13.1)	13.0 (2.0-67.0)	
**Submission to decision, with peer-review comparisons**				
All journals	28	80.9 (55.7)	60.5 (21.0-263.0)	NA
Open access	11	72.9 (51.5)	58.0 (21.0-186.0)	.48
Hybrid/subscription	17	86.1 (59.2)	61.0 (31.0-263.0)	
Highly selective	4	48.0 (15.0)	49.0 (31.0-63.0)	.01
All others	24	86.4 (58.3)	64.0 (21.0-263.0)	
**Submission to final decision, with peer-review comparisons**				
All journals	25	197.7 (67.7)	198.0 (38.0-314.0)	NA
Open access	12	163.5 (57.0)	184.5 (38.0-220.0)	.03
Hybrid/subscription	13	229.3 (62.8)	228.0 (125.0-314.0)	
Highly selective	2	221.0 (66.5)	221.0 (174.0-268.0)	.51
All others	23	195.7 (68.9)	198.0 (38.0-314.0)	
**Submission to publication comparisons**				
All journals	7	164.7 (88.2)	150.0 (73.0-333.0)	NA
Open access	4	123.0 (60.7)	107.5 (73.0-204.0)	.16
Hybrid/subscription	3	220.3 (98.6)	178.0 (150.0-333.0)	
Highly selective	0	NA	NA	NA
All others	7	164.7 (88.2)	150.0 (73.0-333.0)	
**Acceptance to publication, online comparisons**				
All journals	26	31.6 (39.2)	25.5 (2.0-205.0)	NA
Open access	12	40.3 (55.5)	22.5 (2.0-205.0)	.84
Hybrid/subscription	14	24.1 (14.7)	29.0 (3.0-49.0)	
Highly selective	1	37.0 (NA)	37.0 (37.0-37.0)	.50
All other	25	31.4 (39.9)	23.0 (2.0-205.0)	
**Acceptance to publication, print comparisons**				
All journals	5	124.6 (90.9)	105.0 (39.0-252.0)	NA
Open access	0	NA	NA	NA
Hybrid/subscription	5	124.6 (90.9)	105.0 (39.0-252.0)	
Highly selective	2	43.0 (5.7)	43.0 (39.0-47.0)	.06
All others	3	179.0 (73.5)	180.0 (105.0-252.0)	
**Submission to publication online, imputed comparisons** ^b^				
All journals	48	194.0 (78.2)	196.2 (35.0-353.0)	NA
Open access	21	176.8 (63.3)	196.2 (35.0-259.0)	.30
Hybrid/subscription	27	207.0 (85.4)	196.2 (80.0-353.0)	
Highly selective	5	160.1 (87.4)	109.2 (80.0-307.0)	.34
All other	43	197.9 (76.1)	196.2 (35.0-353.0)	

Abbreviations: NA, not applicable; RPT, review and publication time.

^a^ Review and publication timing metrics and open access status were collected from each journal’s primary website or partner website if the journal was owned by a larger publisher. If review and publication timing metrics were reported in 2 places (ie, a year-in-review article, on the About or For Authors page), the metrics listed on the standard webpage were prioritized. If the journal reported a range of timing for a metric, the median time in the analysis was used. All review and publication timing metrics were double-entered, and data discrepancies were resolved by consensus between investigators (K.A.P. and D.M.H.).

^b^ To determine submission to publication time across all journals, imputation for journals that did not report submission to publication time was used and calculated as follows: submission to final decision with peer review plus acceptance to publication; if acceptance to publication was not reported, it was imputed as the median across all reporting journals; if submission to final decision with peer review was not reported, the difference was added between submission to decision with peer review and final decision with peer review (12 days) to submission to first decision with peer review. Six journals did not report any metrics that would allow for partial imputation to calculate submission to publication timing; for these journals, the median across all reporting journals was imputed (196.2 days).

Open access journals had faster final decision (peer-reviewed) times vs hybrid/subscription journals (median [range], 184.5 [38.0-314.0] vs 228.0 [38.0-22.0] days; *P* = .03). Highly selective journals had faster times to first decision without peer-review (median [range], 3.0 [2.0-8.0] vs 13.0 [2.0-67.0] days; *P* < .001) and with peer-review (median [range], 49.0 [31.0-63.0] vs 64.0 [21.0-263.0] days; *P* = .01).

## Discussion

This cross-sectional study shows substantial variation across journals in RPTs reported and time frames. The findings suggest that journals may favor reporting metrics over which they have direct control, eg, initial decision times. We also found that open access journals reported faster review times and highly selective journals reported faster first decisions.

Our findings validate and extend earlier research, documenting substantial variation in RPTs.^4,5,6^ However, most research focused on individual articles within specialized journals. By assessing journal-level RPTs across health policy publications, including all articles within journals, our study may provide a broader understanding of RPT patterns.

The lack of standardized RPT reporting limits transparency. Standardized RPT metrics and reporting across journals might enhance comparisons and help researchers choose which publication may best suit their study and career. The RPT should be included in standardized databases and tracking systems, as is done for other metrics (eg, impact factor).

We used multiple approaches to identify journals that publish on health policy topics but potentially excluded more specialized health policy journals. Our analysis relied on journals’ RPT descriptions because RPTs are not standardized, which may have introduced uncertainty to the extent that journal measurements were inconsistent. Journals often group all publication types together; thus, our results do not relate to research articles alone but represent all types of published articles.

Academic publications are important for the fast dissemination of science and addressing policy challenges.^1,2,3^ Therefore, additional research should explore what factors contribute to publication speed and the tradeoffs necessary to increase publication times.

## Notes

Supplement 1. **eTable.** Journals Included in Study Sample

Supplement 2.
Data Sharing Statement
