# Supplementary material for: Review and Publication Times and Reporting Across Journals on Health Policy
Source: JAMA Netw Open. 2025 May 27;8(5):e2512545. doi: 10.1001/jamanetworkopen.2025.12545 (PMC12117454; doi:10.1001/jamanetworkopen.2025.12545)
Supplement: Supplement 1. — eTable. Journals Included in Study Sample [file jamanetwopen-e2512545-s001.pdf]

# Supplemental Online Content

Phillips KA, Horn DM. Health policy review and publication times and reporting across journals. *JAMA Netw Open*. 2025;8(5):e2512545. doi:10.1001/jamanetworkopen.2025.12545

**eTable.** Journals Included in Study Sample

This supplemental material has been provided by the authors to give readers additional information about their work.

© 2025 Phillips KA et al. *JAMA Network Open*.

## eTable. Journals Included in Study Sample

| | |
|---|---|
| American Journal of Health Economics | International Journal of Technology Assessment in Health Care |
| American Journal of Managed Care | JAMA * |
| American Journal of Public Health | JAMA Health Forum |
| Annual Review of Public Health | JAMA Internal Medicine * |
| Applied Health Economics and Health Policy | Journal of Health Care for the Poor and Underserved |
| Archives of Public Health | Journal of Health Economics |
| BMC Health Services Research | Journal of Health Economics and Outcomes Research |
| BMC Public Health | Journal of Health Equity |
| BMJ Quality & Safety | Journal of Health Politics, Policy and Law |
| British Medical Journal * | Journal of Managed Care & Specialty Pharmacy |
| British Medical Journal Open | Journal of Medical Economics |
| Global Health Research and Policy | Journal of Public Health |
| Health Affairs | Journal of Public Health Policy |
| Health Affairs Scholar | Medical Care |
| Health Economics Review | Medical Care Research and Review |
| Health Economics, Policy and Law | Milbank Quarterly |
| Health Policy | NATURE * |
| Health Policy and Planning | NEJM Catalyst Innovations in Care Delivery |
| Health Policy and Technology | New England Journal of Medicine * |
| Health Policy OPEN | Perspectives in Public Health |
| Health Research Policy and Systems | PLOS One |
| Health Services Research | Public Health |
| Health Systems & Reform | Public Health Reports |
| Healthcare | Public Health Reviews |
| HealthCare-The Journal of Delivery Science and Innovation | Science * |
| Health Economics | Social Science and Medicine |
| Implementation Science | The International Journal of Health Planning and Management |
| INQUIRY: The Journal of Health Care Organization, Provision, and Financing | Value in Health |
| International Journal of Health Policy and Management | |

*Highly Selective: impact factor above 20.0 and acceptance rate below 15%.

© 2025 Phillips KA et al. *JAMA Network Open*.
