# Supplementary material for: Review and Publication Times and Reporting Across Journals on Health Policy
Source: JAMA Netw Open. 2025 May 27;8(5):e2512545. doi: 10.1001/jamanetworkopen.2025.12545 (PMC12117454; doi:10.1001/jamanetworkopen.2025.12545)
Supplement: Supplement 2. — Data Sharing Statement [file jamanetwopen-e2512545-s002.pdf]

Data Sharing Statement

Phillips. Review and Publication Times and Reporting Across Journals on Health Policy. *JAMA Netw Open*. Published May 27, 2025. doi:10.1001/jamanetworkopen.2025.12545

Data
Data available: Yes
Data types: Data (not involving human participants)
How to access data: All data is publicly available
When available: With publication

Supporting Documents
Document types: None

Additional Information
Who can access the data: Anyone
Types of analyses: Any purpose
Mechanisms of data availability: All data is publicly available
